# Comparative Analysis of Visual Performance and Astigmatism Tolerance with Monofocal, Bifocal, and Extended Depth-of-Focus Intraocular Lenses Targeting Slight Myopia

**DOI:** 10.1155/2020/9283021

**Published:** 2020-10-24

**Authors:** Jie Xu, Tianyu Zheng, Yi Lu

**Affiliations:** ^1^Department of Ophthalmology, Eye and ENT Hospital, Fudan University, 83 Fenyang Rd., Shanghai 200031, China; ^2^Eye Institute, Eye and ENT Hospital, Fudan University, Shanghai, China; ^3^Key Laboratory of Myopia, Ministry of Health, Shanghai, China; ^4^Shanghai Key Laboratory of Visual Impairment and Restoration, Shanghai, China

## Abstract

**Purpose:**

To compare the visual performance and astigmatism tolerance of 3 intraocular lens (IOL) groups: monofocal, bifocal, and extended depth-of-focus (EDOF) IOLs targeting slight myopia.

**Methods:**

Overall, there were 60 cataract surgery eyes from 60 patients with implantation of a monofocal, bifocal, or EDOF IOL (20 eyes in each IOL group). The EDOF IOLs targeted slight myopia (−0.25 *D* to −0.75 *D*). Intragroup comparison of visual acuity, defocus curve, objective optical quality, contrast sensitivity, visual function questionnaire scores, patients' overall satisfaction, and the astigmatism tolerance was performed 3 months after surgery.

**Results:**

The EDOF group provided equivalently excellent distance visual outcomes (0.06 ± 0.12) as the monofocal (0.06 ± 0.09) and bifocal (0.03 ± 0.09) groups (*P*=0.554), better intermediate vision than the other 2 groups (*P* < 0.05), and similarly satisfactory near visual outcomes (0.23 ± 0.16 at 20 cm, and 0.17 ± 0.14 at 33 cm) as the bifocal group (0.28 ± 0.14 at 20 cm and 0.08 ± 0.10 at 33 cm) (*P* > 0.05). The contrast sensitivity of EDOF IOL was slightly decreased compared to that of monofocal IOL, but it was better than that of bifocal IOL. The EDOF group showed significantly higher satisfaction than the bifocal group did when preoperative corneal astigmatism was 0.75 *D* or greater (*P*=0.009). A significant negative correlation between the corneal astigmatism and patient satisfaction was observed in only the bifocal group.

**Conclusions:**

The EDOF IOLs targeting slight myopia offered satisfactory visual outcomes at an extended range from far to near distances. The EDOF and monofocal IOLs showed a better tolerance to astigmatism than did the bifocal IOL.

## 1. Introduction

New IOL designs are focused on improving both distance and intermediate vision, while maintaining an adequate near vision, as well as minimizing the dysphotopsia phenomena associated with traditional multifocal IOLs [1, 2]. The extended depth-of-focus (EDOF) IOL was recently developed based on the elongated focus, thereby minimizing the dysphotopsia phenomena induced by multifocality and providing better optical quality on the whole addition range [2, 3]. However, the EDOF IOL resulted in worse outcomes for near vision compared to those for far and intermediate vision [2, 4–6]. A recently published study demonstrated that the eyes with implantation of EDOF IOL aimed for −0.75 *D* myopia provided better uncorrected near vision and similar uncorrected intermediate vision compared with the emmetropia eyes and maintained excellent distance vision outcomes at the same time [7]. It is speculated that, in order to achieve satisfactory near vision, slight myopia following the implantation of this IOL may be attempted. To date, no studies have compared the visual performance of EDOF IOL, when targeting slight myopia, with that of monofocal IOL and bifocal IOL.

Additionally, one major concern is the significant impact of corneal astigmatism on postoperative visual outcomes. Previous studies have demonstrated that the multifocal IOLs presented a worse tolerance to astigmatism than that of monofocal IOL [8, 9]. However, the diverse impact of preoperative corneal astigmatism on visual performance among monofocal IOLs, bifocal IOLs, and EDOF IOLs has not been analyzed.

The main purpose of this study was to compare visual performance and astigmatism tolerance of the new EDOF IOL with those of the monofocal IOL and bifocal IOL and to evaluate the benefits of intended slight myopia in the EDOF IOL.

## 2. Methods

### 2.1. Patients

This study was approved by the ethics committee of the Eye and Ear, Nose, and Throat (ENT) Hospital of Fudan University and was conducted according to the principles of the Declaration of Helsinki. This prospective nonrandomized comparative clinical study enrolled patients who had cataract surgery with implantation of 1 of the 3 following IOL models: Tecnis monofocal (ZCB00, Abbott Medical Optics, Santa Ana, CA, USA), Tecnis +4.0D bifocal (ZMB00, Abbott Medical Optics, Santa Ana, CA, USA), or Tecnis EDOF (ZXR00, Abbott Medical Optics, Santa Ana, CA, USA) IOLs. All patients provided informed consent. The inclusion criteria were healthy eyes besides having age-related cataract, preexisting total corneal astigmatism <1.50 *D*, and an axial length (AL) of 20 mm to 26 mm. The exclusion criteria included presence of any other eye pathology or neuropathy. We further divided the patients of each IOL type into 2 subgroups (with total corneal astigmatism of <0.75 *D* or ≥0.75 *D*) when analyzing the astigmatism tolerance for the 3 IOL groups.

### 2.2. Preoperative Examinations

Preoperatively, all patients underwent comprehensive ophthalmologic examinations, including measurements of uncorrected visual acuity, fundus examination, assessment with IOL Master 500 (Carl Zeiss AG, Oberkochen, Germany), and B mode ultrasound scan. The OPD-Scan III aberrometer (Nidek Co, Ltd, Gamagori, Japan) provided information on pupil size, as well as the alpha angle and kappa angle, under photopic and mesopic lighting conditions. Total corneal astigmatism was measured by corneal topography (Pentacam HR; Oculus Optikgeräte, Wetzlar, Germany).

### 2.3. Intraocular Lens Power and Surgical Technique

The IOL power and predicted postoperative refractive errors were based on biometric data measured with the IOL Master 500. In the IOL power calculations, the SRK-T formula was used for all eyes with an AL of 22.0∼26.0 mm, and the Hoffer *Q* formula was used for the eyes with an AL of 20.0∼22.0 mm. The IOL power was selected to achieve slight myopia (−0.25 *D* to −0.75 *D*) in participants of the EDOF group, whereas all the eyes of patients in the monofocal and bifocal groups targeted emmetropia.

All surgeries were performed by a single experienced surgeon (YL) using a standard sutureless phacoemulsification technique. A 2.6 mm clear corneal incision was made at the steep meridian in all patients. The IOL was implanted in the capsular bag and adjusted to the center.

### 2.4. Three-Month Postoperative Examinations

Postoperative follow-up examinations were performed at 3 months after surgery. The following tests were performed: measurement of uncorrected distance visual acuity (UDVA), best corrected distance visual acuity (CDVA), uncorrected intermediate visual acuity (UIVA) and distance-corrected intermediate visual acuity (DCIVA) at 80 cm, and uncorrected near visual acuity (UNVA) and distance-corrected near visual acuity (DCNVA) at 20 cm and 33 cm. Defocus curves were performed with best distance correction. Different levels of defocus were introduced in 0.50 *D* steps from +2.00 to −4.00 *D*.

The objective optical quality, including the modulation transfer function (MTF) and point spread function (PSF) was evaluated with the OPD-Scan III aberrometer. A metric for the MTF is provided as the area ratio (AR) value, while the PSF was analyzed using the Strehl ratio (SR) value. MTF (total) and SR (total) values were calculated from total aberrations in one measurement, provided by the OPD-Scan III aberrometer, while the MTF (HO) and SR (HO) values were calculated from only HOAs corresponding to the MTF and SR values with the correction of refractive errors. All of these abovementioned measurements were reported at 3.0 mm pupil diameters (PD).

Subjective CS was measured with the best distance correction using the Functional Acuity Contrast Test (FACT) of the Optec 6500 view-in test system (Stereo Optical Co., Inc., Chicago, Illinois, USA) at spatial frequencies of 1.5, 3, 6, 12, and 18 cpd under photopic (85 candelas/square meter (cd/m^2^)) and mesopic (3 cd/m^2^) conditions with and without glare. Absolute values of log10 CS were obtained for each spatial frequency.

### 2.5. Questionnaire

At the end of the 3-month follow-up, a subjective questionnaire regarding visual perceptions for various activities, spectacle dependence, dysphotopsia symptoms, and patients' overall satisfaction was administered to all patients. Visual perceptions were assessed with the Visual Function Index-14 (VF-14) questionnaire. The maximum score was 4 and the minimal score was 0. In addition to the total VF-14 score, 2 additional scores were calculated: the distance vision VF score (items 4, 5, 6, 10, 12, 13, and 14), and the near vision VF score (items 1, 2, 3, 7, 8, 9, and 11) [10]. All VF-14 scores were converted into a 100-point scale, in which higher scores indicated better visual perceptions [10]. In addition, two direct 4-scale Likert-type questions (1, severe; 2, moderate; 3, slight; 4, none) that pertained to the subjective perception of glare and halos were included. The spectacle dependence for total vision activities was also assessed by direct 4-scale Likert-type questions (1, always; 2, most of the time; 3, sometimes; 4, never). Lastly, the patients were asked to grade their level of overall satisfaction of using the 5-point Likert scale (1, very dissatisfied; 2, dissatisfied; 3, neither satisfied nor dissatisfied; 4, satisfied; 5, very satisfied).

### 2.6. Statistical Analysis

The statistical analysis was performed using SPSS 22.0 (IBM Corp., Armonk, NY, USA). Normality of the data samples was evaluated by the Kolmogorov–Smirnov test. If parametric analysis was possible, a 1-way analysis of variance (ANOVA) with Bonferroni post hoc analysis was used to compare the parameters analyzed among the IOLs. Otherwise, the Kruskal–Wallis test was used to assess the significance of such differences. The Mann–Whitney *U* test was used when comparing between the 2 astigmatism subgroups of each IOL type. The relationships between the preoperative corneal astigmatism and overall satisfaction scores were assessed using Spearman's correlation coefficients. The *X*^2^ test was used to examine differences in sex distribution and proportions of spectacle independence and dysphotopsia perception, and Bonferroni adjustment was applied for multiple comparisons of categorical variables among the 3 groups. A *P* < 0.05 was considered statistically significant.

## 3. Results

Overall, 60 eyes from 60 participants were included in this study, and 20 eyes were included in each IOL group.  Table 1 summarizes the preoperative patient characteristics in the 3 groups. No significant intragroup differences were identified in the baseline information, except for target refraction. The EDOF group, in which slight myopia was targeted, presented a significantly higher negative target refraction than that of the other 2 groups (*P* < 0.001).

### 3.1. Visual Outcomes

**Figure 1:**
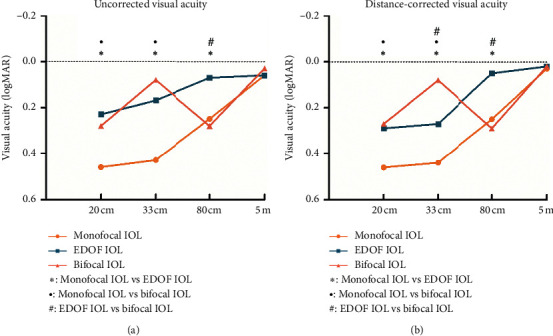
Comparison of the mean visual acuity at all distances among the 3 intraocular lens (IOL) groups (^*∗*^ = statistically significant difference between the monofocal group and the extended range of vision (EDOF) group [*P* < 0.05]; ^●^ = statistically significant difference between the monofocal group and the bifocal group [*P* < 0.05]; ^#^ = statistically significant difference between the EDOF group and the bifocal group [*P* < 0.05]; logMAR = logarithm of minimum angle of resolution).

Table 2 and Figure 1 show the postoperative visual outcomes in the 3 IOL groups. Consistent with the expected refractive errors, the EDOF group obtained the highest negative diopter (−0.83 ± 0.52 D) (*P*=0.003 and *P* < 0.001 compared to that of the monofocal and bifocal groups, respectively).

All 3 groups achieved excellent distant vision, with mean UDVAs of 0.06 ± 0.09 logMAR, 0.06 ± 0.12 logMAR, and 0.03 ± 0.09 logMAR, in the monofocal IOL, EDOF IOL, and bifocal IOL groups, respectively. No statistically significant differences in UDVA and CDVA were observed among the 3 groups. However, in the EDOF IOL group, the UDVA was significantly worse than the CDVA (*P*=0.020).

Regarding the UIVA and DCIVA values, the differences among the 3 groups reached statistical significance, with the EDOF IOL group showing significantly better results than those of the monofocal IOL (*P*=0.003 for UIVA and *P*=0.001 for DCIVA) and bifocal IOL (*P*=0.001 for both UIVA and DCIVA) groups. No statistically significant difference was observed between the monofocal and bifocal IOL groups.

The UNVA values were significantly better in the EDOF IOL and bifocal IOL groups than they were in the monofocal IOL group (all *P* < 0.01). No statistically significant differences in UNVA between the bifocal IOL and EDOF IOL groups were identified (*P*=1.000 at 20 cm and *P*=0.259 at 33 cm), although the average logMAR value (33 cm) in bifocal group was lower (better) than that in the EDOF group. Likewise, the DCNVA (20 cm) results were similar between the EDOF IOL and bifocal IOL groups (*P*=1.000), both of which were significantly better than those of the monofocal IOL group (*P*=0.002 and *P* < 0.001, respectively). In terms of the DCNVA (33 cm), the bifocal IOL had the best outcomes when compared with the EDOF and monofocal IOL groups (*P*=0.002 and *P* < 0.001, respectively).

### 3.2. Defocus Curve

**Figure 2:**
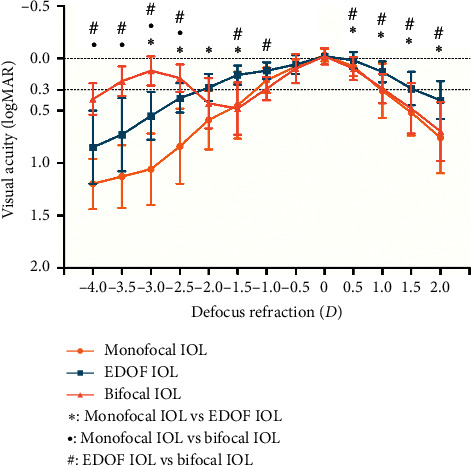
The defocus curves in the 3 intraocular lens (IOL) groups. Bars around data points correspond to the standard deviation (SD) (^*∗*^ = statistically significant difference between the monofocal group and the extended range of vision (EDOF) group [*P* < 0.05]; ^●^ = statistically significant difference between the monofocal group and the bifocal group [*P* < 0.05]; ^#^ = statistically significant difference between the EDOF group and the bifocal group [*P* < 0.05]; logMAR = logarithm of minimum angle of resolution).

Figure 2 shows the distance-corrected defocus curves obtained with the 3 IOLs. Visual acuity was significantly better in the bifocal IOL group than that in the EDOF and monofocal groups for defocus vergences from −4.0 *D* to −2.50 *D*. Additionally, a significantly better visual acuity was obtained in the EDOF group than that in the bifocal and monofocal groups at positive defocus ranging from +0.5 *D* to +2.0 *D*. Visual acuity remained better than 0.3 logMAR throughout 0 *D* to +1.5 *D* in patients in the EDOF IOL group.

### 3.3. Objective Optical Quality Outcomes

**Figure 3:**
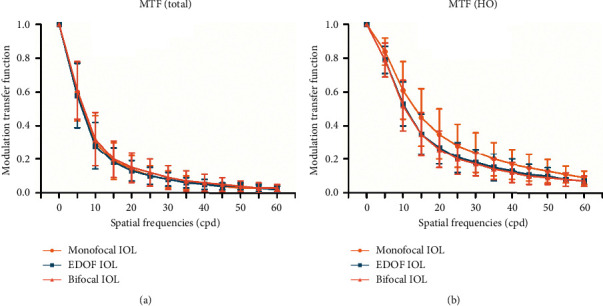
Comparison of the mean total (ocular) modulation transfer function (MTF) values (a) and mean MTF (HO) values (b) among the 3 intraocular lens (IOL) groups at different spatial frequencies. No significant difference was observed among the 3 IOL groups for any of the spatial frequencies. Error bars represent the standard deviation (SD) of the mean (total: indicates data calculated from total aberrations; HO: data calculated from only high-order aberrations; EDOF = extended range of vision).

An intragroup comparison of the MTF and PSF values is provided in Figure 3 and Table S1. No statistically significant differences between groups were obtained, though the MTF (HO) values were insignificantly higher in monofocal group than the other 2 groups.

### 3.4. Subjective Contrast Sensitivity Outcomes

**Figure 4:**
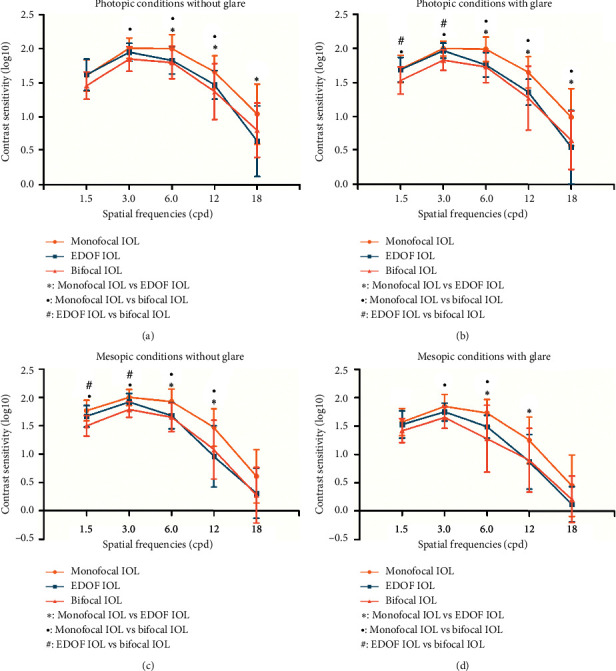
Comparison of the mean contrast sensitivity (CS) values among the 3 intraocular lens (IOL) groups at different spatial frequencies under photopic conditions without glare (a), mean CS values under photopic conditions with glare (b), mean CS values under mesopic conditions without glare (c), and mean CS values under mesopic conditions with glare (d). Error bars represent the standard deviation (SD) of the mean (^●^ = statistically significant difference between the monofocal group and the extended-range-of-vision (EDOF) group [*P* < 0.05]; ^#^ = statistically significant difference between the monofocal group and the bifocal group [*P* < 0.05]; ^*∗*^ = statistically significant difference between the EDOF group and the bifocal group [*P* < 0.05]).

The mean values of log10 CS after implantation of the 3 IOLs are plotted as a series of CS functions in Figure 4. The monofocal group performed best in the assessment of CS. When compared with the bifocal IOL, the monofocal IOL presented higher CS results at any lighting condition and spatial frequency with or without glare, with significant differences in all except for 1.5 and 18 cpd under photopic conditions without glare, 18 cpd under mesopic conditions without glare, and 1.5, 12, and 18 cpd under mesopic conditions with glare. When compared with the EDOF group, the monofocal group presented a significantly better CS under photopic conditions at 6, 12, and 18 cpd and under mesopic conditions at 6 and 12 cpd. In addition, significantly higher CS values were obtained with the EDOF IOL than those with the bifocal IOL at low frequencies (1.5 and 3 cpd) under photopic conditions with glare and under mesopic conditions without glare.

### 3.5. Questionnaire

The VF-14 scores, level of spectacle dependence, complaints of dysphotopsia, and overall patient satisfaction are presented in Table 3. No significant differences in VF items that pertained to distance (*P*=0.070) vision were detected among the 3 groups. Regarding the total VF-14 scores and the VF items related to near vision, both the EDOF group and bifocal group presented significantly better results than did the monofocal group (*P* < 0.001), whereas no significant difference was observed between the EDOF and bifocal groups. The scores of spectacle dependence for the EDOF group and bifocal groups were similar (*P*=1.000), and both were better than the score for the monofocal group (*P* < 0.001). A significantly higher proportion of spectacle-free patients was observed in the EDOF (60%) and bifocal IOL groups (65%) than that in the monofocal IOL group (10%) (*P* < 0.05), while no significant difference was identified between the EDOF and bifocal groups (*P* > 0.05). Next, the scores for glare or halos perception showed no significant difference among the 3 groups. The overall patient satisfaction scores were the highest in the EDOF IOL group, followed by the bifocal IOL group. The monofocal IOL had the lowest scores. However, a significant difference in the scores was obtained only when comparing the EDOF IOL group with the monofocal IOL group (*P*=0.007).

### 3.6. Astigmatism Tolerance

In each IOL group, the patients were divided into two subgroups, as follows, based on preoperative total corneal astigmatism: low astigmatism subgroup (less than 0.75 *D*, 11 eyes in the monofocal group, 10 eyes in the EDOF group, and 11 eyes in the bifocal group) and high astigmatism subgroup (equal to or greater 0.75 *D*, 9 eyes in the monofocal group, 10 eyes in the EDOF group, and 9 eyes in the bifocal group).

**Figure 5:**
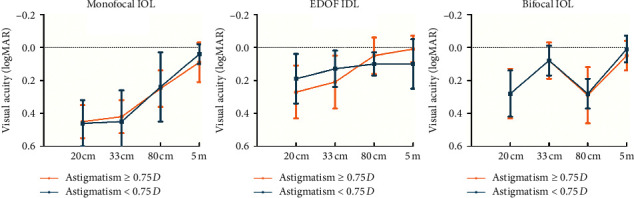
Comparison of the mean uncorrected visual acuity, from far to near, between the two astigmatism subgroups in the 3 intraocular lens (IOL) groups. No significant difference was observed between the two astigmatism subgroups in any of the 3 IOL groups. Error bars represent the standard deviation (SD) of the mean (logMAR = logarithm of minimum angle of resolution).

No significant differences were found in the mean uncorrected visual acuity, visual quality, VF-14 scores, level of spectacle dependence, or the perception of dysphotopsia between the 2 astigmatism subgroups in each IOL group (Figure 5, Table S2, Figure S1). However, the bifocal group presented an insignificantly slight deterioration of visual function for near vision and glare perception in the high astigmatism subgroup (*P*=0.261 and *P*=0.824, respectively) (Table S2).

**Figure 6:**
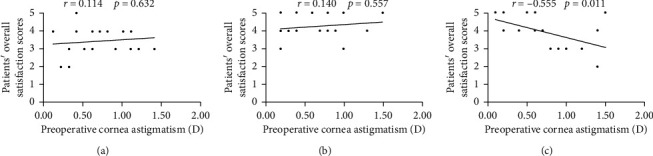
Correlations between the overall patient satisfaction score and preoperative corneal astigmatism in the 3 intraocular lens (IOL) groups. (a) Monofocal IOL group (Spearman's correlation coefficient: *r*=0.114, *P*=0.632). (b) Extended range of vision (EDOF) IOL group (Spearman's correlation coefficient: *r*=0.140, *P*=0.557). (c) Bifocal IOL group (Spearman's correlation coefficient: *r*=−0.555, *P*=0.011).

However, in the bifocal group, the overall satisfaction scores of the high astigmatism subgroup were significantly poorer than those of the low astigmatism subgroup, with the mean score reducing from 4.45 to 3.22 (*P*=0.002, Table S2). In contrast, for the monofocal group and EDOF group, the satisfaction scores in the high astigmatism subgroup (satisfaction scores = 3.56 for the monofocal group and 4.30 for the EDOF group) were almost the same as those in the low astigmatism subgroups (satisfaction scores = 3.36 for the monofocal group and 4.20 for the EDOF group, *P*=0.656 and *P*=0.739, respectively) (Table S2). Furthermore, the satisfaction scores were negatively correlated with preoperative corneal astigmatism in only the bifocal group (*r*=−0.555, *P*=0.011) (Figure 6).

The comparisons of visual outcomes and other quality of vision parameters among the 3 IOLs did not change a lot when the corneal astigmatism was larger (Figures S2 and S3 and Table S3). However, the comparison of patient satisfaction among the 3 IOLs was significantly different between the 2 astigmatism subgroups. In the low astigmatism subgroups, the satisfaction scores in the bifocal group (4.45) and EDOF group (4.20) were similar (*P*=1.000), and both were better than those of the monofocal group (3.36). However, in the high astigmatism subgroups, the satisfaction scores of the bifocal group (3.22) were the lowest, and a significant difference was identified when it was compared with those of the EDOF group (4.30) (*P*=0.009) (Table S3).

## 4. Discussion

The current study is the first to provide a comparison of the visual performance and astigmatism tolerance of the EDOF IOL, when targeting slight myopia, with those of monofocal IOL and bifocal IOL.

As previous studies have demonstrated, the EDOF IOL targeting for emmetropia could obtain a similar distance visual acuity as the monofocal IOL and multifocal IOL [4, 11, 12]. In our study, although the EDOF IOL was with targeted slight myopia, the EDOF IOL also provided an equivalently excellent distance visual acuity to that in the other 2 groups. Similarly, Ganesh et al. demonstrated that, when patients implanted with the EDOF IOLs targeted myopia to be within −0.75 *D*, they could still maintain an adequate uncorrected distance vision [7]. The good tolerability of the EDOF IOL to the postoperative myopic refractive errors may be explained by the good performance of the EDOF IOL at the positive defocus. In the current study, the EDOF group still maintained a satisfactory visual acuity (better than 0.3 logMAR) for defocus vergences from 0 *D* to +1.5 *D* and was significantly better than the other 2 groups. However, the UDVA of the EDOF group was significantly worse than its CDVA, indicating that the targeted slight myopia may still have certain negative impacts on UDVA.

Pedrotti et al. [2] and Yoo et al. [6] demonstrated that the EDOF IOL, compared with the monofocal IOL and bifocal IOL, presented the best outcomes at intermediate distance. Consistent with these studies, we observed in our study that the significant superiority of EDOF IOL in terms of intermediate vision over monofocal and bifocal IOLs was maintained when targeting slight myopia.

A major concern of the EDOF IOL was the relatively worse near vision that it produced compared to that of the traditional bifocal IOL [2, 5, 13]. With best distance correction, our study also showed a significantly worse DCNVA (33 cm) and a significantly worse visual acuity at the defocus curve, ranging from −4.0 *D* (25 cm) to −2.5 *D* (40 cm), with the EDOF IOL than that with the bifocal IOL. However, we observed that the uncorrected near vision of the EDOF IOL targeting slight myopia was similarly satisfactory as that of the bifocal group. Our findings confirm the benefits of an intended postoperative slight myopia in the EDOF IOL, which may be attempted to achieve satisfactory outcomes for near vision.

In accordance with the VF-14 scores, all 3 IOL groups demonstrated equivalently excellent visual functions at distance range. Patients in both the EDOF and bifocal IOL groups obtained statistically better visual function for near vision and overall visual function than did the patients in the monofocal group. Accordingly, patients in both the EDOF and bifocal IOL groups are more spectacle-independent than those in the monofocal group are. These proportions of spectacle-free patients in this study were comparable to those of other studies with monofocal IOL, EDOF IOL, and bifocal IOL [14, 15].

In the objective optical quality evaluation, the mean MTF (HO) values of the monofocal IOL had an insignificantly advantage over those of the EDOF group and bifocal group. Consistently, with regard to the subjective optical quality, the monofocal IOL presented better CS results than did the EDOF IOL and bifocal IOL. The CS of the EDOF IOL was lower than that of the monofocal IOL at the medium and high spatial frequencies, but it was as good as that of the monofocal IOL at low spatial frequencies. Similar to our results, previous studies have reported better CS values with the monofocal and EDOF IOL than those with the multifocal IOL [2, 16, 17].

For the subjective perception of dysphotopsia phenomena, both the EDOF group and bifocal group perceived no significantly more glare or halos than did the monofocal group, which is consistent with the similar PSF values shown among the 3 groups in the current study. In contradiction to our results, Puell et al. and Monaco et al. found the patients of multifocal IOL and EDOF IOL group had a higher incidence of visual side effects than the monofocal group [4, 18]. However, Pedrotti et al. also detected no statistically significant differences regarding dysphotopsia perception among the 3 IOL groups [2]. The reasons for this result may be the differences in IOL models, follow-up times, and sample sizes [19].

The overall satisfaction scores were the highest in the EDOF group, although a significant difference was only identified when compared with those of the monofocal group. Consistent with this finding, in a previous study by Sachdev et al., patients implanted with the EDOF IOL rated their satisfaction very high, and 96 percent of the patients agreed to choosing the same IOL again and recommending it to their friends and family [20].

In his study, when the preoperative corneal astigmatism reached 0.75 *D* or greater, the overall satisfaction scores became significantly poorer in only the bifocal IOL group. Furthermore, correlation analysis confirmed that the corneal astigmatism negatively correlated with the satisfaction scores only in the bifocal group. This finding may be due to the slight deterioration of visual function for near vision and glare phenomenon in the bifocal group when the corneal astigmatism was 0.75 *D* or greater (Table S2). Similar to our findings, visual acuity and dysphotopsia phenomenon of multifocal IOLs were demonstrated to be compromised by astigmatisms greater than 1.00 *D* [8, 9, 21], while the monofocal and EDOF IOLs were less compromised [8, 9, 22]. Carones et al. also demonstrated that patients showed less dissatisfaction to the EDOF IOL than to the multifocal IOLs when the astigmatism was 0.75 *D* or greater [22]. These previous studies and our findings indicated that the bifocal IOLs were less tolerant to corneal astigmatism than were the EDOF IOLs and monofocal IOLs.

This study has some limitations that should be mentioned. First, the patients were not randomized to the 3 IOL groups. The EDOF or the bifocal IOLs were implanted in the eyes of patients requesting spectacle independence. Another limitation is the limited sample size, especially when comparing the astigmatism tolerance. Further studies with a larger sample size are required to validate the results of this study.

## 5. Conclusion

In conclusion, the current study demonstrates that the EDOF IOL, when targeting slight myopia, maintains an extended range of sharp vision, from far to intermediate distances and provides a similarly satisfactory near visual acuity as that of the bifocal IOL. The EDOF group also provided better intermediate vision and low-frequency CS than that of the bifocal group. Furthermore, the EDOF IOL and monofocal IOL showed a significantly better tolerance to astigmatism than did the bifocal IOLs.

## Figures and Tables

**Table 1:** Intragroup comparison of baseline characteristics in the 3 IOL groups.

Parameter (mean ± SD)	Monofocal IOL (*A*)	EDOF IOL (*B*)	Bifocal IOL (*C*)	*P* value
Age (y)	64.65 ± 7.75	64.45 ± 14.68	58.85 ± 11.32	0.107
Sex, n (%)				0.535
Female	13 (65%)	10 (50%)	13 (65%)	
Male	7 (35%)	10 (50%)	7 (35%)	
Preoperative UDVA (logMAR)	0.75 ± 0.39	0.61 ± 0.31	0.73 ± 0.42	0.495
Cornea astigmatism	0.71 ± 0.37	0.67 ± 0.36	0.73 ± 0.40	0.860
ACD	3.15 ± 0.29	3.30 ± 0.40	3.27 ± 0.28	0.330
AL (mm)	23.85 ± 0.89	23.97 ± 1.10	23.37 ± 0.79	0.115
IOL power (D)	21.30 ± 1.64	20.83 ± 3.00	21.68 ± 2.28	0.529
Expected refractive error (D)	−0.16 ± 0.05	−0.45 ± 0.13	0.13 ± 0.09	<0.001^*∗*^
				A–B <0.001^*∗*^
				A–C <0.001^*∗*^
				B–C <0.001^*∗*^
Photopic pupil size (mm)	3.07 ± 0.43	2.90 ± 0.59	3.03 ± 0.37	0.497
Mesopic pupil size (mm)	5.07 ± 1.08	4.80 ± 1.07	5.03 ± 1.01	0.677
Photopic angle *κ*	0.31 ± 0.16	0.27 ± 0.10	0.29 ± 0.11	0.526
Mesopic angle *κ*	0.34 ± 0.18	0.28 ± 0.11	0.32 ± 0.15	0.500
Photopic angle *α*	0.51 ± 0.13	0.47 ± 0.19	0.51 ± 0.14	0.732
Mesopic angle *α*	0.48 ± 0.18	0.45 ± 0.15	0.50 ± 0.17	0.647

IOL = intraocular lens, EDOF = extend depth-of-focus, SD = standard deviation, ACD = anterior chamber depth, AL = axial length, UDVA = uncorrected visual acuity, and logMAR = logarithm of the minimum angle of resolution. ^*∗*^Statistically significant (*P* < 0.05).

**Table 2:** Intragroup comparison of visual outcomes in the 3 IOL groups.

Visual acuity (LogMAR)	Monofocal IOL (*A*)	EDOF IOL (*B*)	Bifocal IOL (*C*)	*P* value
UDVA (5 m)				0.554
Mean ± SD	0.06 ± 0.09	0.06 ± 0.12	0.03 ± 0.09	
Median (25% and 75% IR)	0.08 (0.00, 0.10)	0.00 (0.00, 0.19)	0.00 (−0.06, 0.10)	
UIVA (80 cm)				<0.001^*∗*^
Mean ± SD	0.25 ± 0.17	0.07 ± 0.09	0.28 ± 0.13	A–B 0.003^*∗*^
Median (25% and 75% IR)	0.20 (0.10, 0.39)	0.10 (0.00, 0.10)	0.30 (0.20, 0.40)	A–C 0.790
				B–C <0.001^*∗*^
UNVA (33 cm)				<0.001^*∗*^
Mean ± SD	0.43 ± 0.15	0.17 ± 0.14	0.08 ± 0.10	A–B <0.001^*∗*^
Median (25% and 75% IR)	0.40 (0.40, 0.57)	0.20 (0.03, 0.20)	0.05 (0.00, 0.18)	A–C <0.001^*∗*^
				B–C 0.259
UNVA (20 cm)				<0.001^*∗*^
Mean ± SD	0.46 ± 0.12	0.23 ± 0.16	0.28 ± 0.14	A–B <0.001^*∗*^
Median (25% and 75% IR)	0.45 (0.40, 0.57)	0.20 (0.10, 0.30)	0.30 (0.20, 0.40)	A–C 0.002^*∗*^
				B–C 1.000
SE				<0.001^*∗*^
Mean ± SD	−0.18 ± 0.42	−0.83 ± 0.52	0.14 ± 0.39	A–B 0.003^*∗*^
Median (25% and 75% IR)	−0.13 (−0.47, 0.13)	−0.75 (−1.10, −0.41)	0.13 (0.00, 0.35)	A–C 0.108
				B–C <0.001^*∗*^
CDVA (5 m)				0.959
Mean ± SD	−0.03 ± 0.06	−0.02 ± 0.08	−0.02 ± 0.08	
Median (25% and 75% IR)	0.00 (−0.08, 0.00)	0.00 (−0.08, 0.00)	0.00 (−0.08, 0.00)	
DCIVA (80 cm)				<0.001^*∗*^
Mean ± SD	0.25 ± 0.14	0.05 ± 0.11	0.29 ± 0.09	A–B <0.001^*∗*^
Median (25% and 75% IR)	0.25 (0.20, 0.30)	0.00 (−0.08, 0.18)	0.30 (0.23, 0.40)	A–C 0.636
				B–C <0.001^*∗*^
DCNVA (33 cm)				<0.001^*∗*^
Mean ± SD	0.44 ± 0.13	0.27 ± 0.12	0.08 ± 0.09	A–B 0.019^*∗*^
Median (25% and 75% IR)	0.45 (0.30, 0.49)	0.30 (0.20, 0.38)	0.05 (0.00, 0.20)	A–C <0.001^*∗*^
				B–C 0.002^*∗*^
DCNVA (20 cm)				<0.001^*∗*^
Mean ± SD	0.46 ± 0.15	0.29 ± 0.13	0.27 ± 0.12	A–B 0.002^*∗*^
Median (25% and 75% IR)	0.49 (0.33, 0.49)	0.30 (0.20, 0.38)	0.20 (0.20, 0.40)	A–C <0.001^*∗*^
				B–C 1.000

IOL = intraocular lens, EDOF = extend depth-of-focus, UDVA = uncorrected visual acuity, UIVA = uncorrected intermediate visual acuity, UNVA = uncorrected near visual acuity, CDVA = best corrected distance visual acuity, DCIVA = distance-corrected intermediate visual, DCNVA = distance-corrected near visual acuity, SD = standard deviation, IR = interquartile ranges, logMAR = logarithm of the minimum angle of resolution, and SE = spherical equivalent. ^*∗*^Statistically significant (*P* < 0.05).

**Table 3:** Intragroup comparison of visual function questionnaire scores and patients' overall satisfaction.

Parameter	Monofocal IOL *(A)*	EDOF IOL *(B)*	Bifocal IOL *(C)*	*P* value
Total VF-14 score				<0.001^*∗*^
Mean ± SD	78.67 ± 9.84	95.38 ± 5.56	92.01 ± 7.12	A–B <0.001^*∗*^
Median (25% and 75% IR)	80.20 (71.87, 85.44)	97.02 (92.67, 100.00)	92.78 (87.50, 98.21)	A–C <0.001^*∗*^
				B–C 0.589
VF-14 (distance vision)				0.070
Mean ± SD	95.19 ± 7.90	99.11 ± 2.56	96.85 ± 5.07	
Median (25% and 75% IR)	100 (93.40, 100.00)	100.00 (100.00, 100.00)	100.00 (95.83, 100.00)	
VF-14 (near vision)				<0.001^*∗*^
Mean ± SD	63.51 ± 14.49	91.94 ± 9.31	87.44 ± 10.08	A–B <0.001^*∗*^
Median (25% and 75% IR)	65.48 (51.04, 75.89)	94.65 (86.16, 100.00)	88.40 (79.46, 96.43)	A–C <0.001^*∗*^
				B–C 0.637
Spectacle dependence				<0.001^*∗*^
Mean ± SD	2.05 ± 1.05	3.45 ± 0.76	3.55 ± 0.76	A–B <0.001^*∗*^
Median (25% and 75% IR)	2.00 (1.00, 3.00)	4.00 (3.00, 4.00)	4.00 (3.00, 4.00)	A–C <0.001^*∗*^
				B–C 1.000
SFP	10% (2/20)	60% (12/20)	65% (13/20)	0.001^*∗*^
				A–B <0.05^*∗*^
				A–C <0.05^*∗*^
				B–C >0.05
Glare				0.314
Mean ± SD	3.35 ± 0.81	3.35 ± 0.81	2.85 ± 1.18	
Median (25% and 75% IR)	4.00 (3.00, 4.00)	4.00 (3.00, 4.00)	3.00 (2.00, 4.00)	
Halos				0.464
Mean ± SD	3.65 ± 0.67	3.60 ± 0.60	3.25 ± 1.07	
Median (25% and 75% IR)	4.00 (3.25, 4.00)	4.00 (3.00, 4.00)	4.00 (2.25, 4.00)	
Percent of patients reporting glare	45% (9/20)	45% (9/20)	55% (11/20)	0.766
Percent of patients reporting halos	25% (5/20)	35% (7/20)	40% (8/20)	0.592
Satisfaction score				0.010^*∗*^
Mean ± SD	3.45 ± 0.76	4.25 ± 0.64	3.90 ± 0.91	A–B 0.007^*∗*^
Median (25% and 75% IR)	3.50 (3.00, 4.00)	4.00 (4.00, 5.00)	4.00 (3.00, 5.00)	A–C 0.264
				B–C 0.551

For VF-14 questionnaire, the maximum score was 4 and the minimal score was 0. All VF-14 scores were converted into a 100-point scale, in which higher scores indicated better visual perceptions. The subjective perception of glare and halos was assessed by two direct 4-scale Likert-type questions (1-severe; 2-moderate; 3-slight; 4-none). The spectacle dependence for total vision activities was also assessed by direct 4-scale Likert-type questions (1-always; 2-most of the time; 3-sometimes; 4-never). The grade of the overall satisfaction used the 5-point Likert scale (1-very dissatisfied; 2-dissatisfied; 3-neither satisfied nor dissatisfied; 4- satisfied; 5-very satisfied). IOL = intraocular lens, EDOF = extend depth-of-focus, VF-14 = visual function index-14, SFP = spectacle-free patients, SD = standard deviation, IR = interquartile ranges, and NA = not available. ^*∗*^Statistically significant (*P* < 0.05).

## Data Availability

The data used to support the findings of this study are available from the corresponding author upon request.

## Supplementary Materials

Supplementary Materials Figure S1: comparison of the mean total (ocular) modulation transfer function (MTF) values between the two astigmatism subgroups of the 3 intraocular lens (IOL) groups. Figure S2: comparison of the mean uncorrected visual acuities at all distances among the 3 intraocular lens (IOL) groups. Figure S3: comparison of the mean total (ocular) modulation transfer function (MTF) values among the 3 intraocular lens (IOL) groups. Table S1: intragroup comparison of objective optical quality outcomes. Table S2: comparison of objective optical quality and visual function questionnaire scores between the 2 astigmatism subgroups in the 3 IOL groups. Table S3: comparison of objective optical quality and visual function questionnaire scores among the 3 IOL types with various preoperative cornea astigmatism.
