# Supplementary material for: Comparative Analysis of Visual Performance and Astigmatism Tolerance with Monofocal, Bifocal, and Extended Depth-of-Focus Intraocular Lenses Targeting Slight Myopia
Source: J Ophthalmol. 2020 Oct 24;2020:9283021. doi: 10.1155/2020/9283021 (PMC7607280; doi:10.1155/2020/9283021)
Supplement: Supplementary Materials — Figure S1: comparison of the mean total (ocular) modulation transfer function (MTF) values between the two astigmatism subgroups of the 3 intraocular lens (IOL) groups. Figure S2: comparison of the mean uncorrected visual acuities at all distances among the 3 intraocular lens (IOL) groups. Figure S3: comparison of the mean total (ocular) modulation transfer function (MTF) values among the 3 intraocular lens (IOL) groups. Table S1: intragroup comparison of objective optical quality outcomes. Table S2: comparison of objective optical quality and visual function questionnaire scores between the 2 astigmatism subgroups in the 3 IOL groups. Table S3: comparison of objective optical quality and visual function questionnaire scores among the 3 IOL types with various preoperative cornea astigmatism. [file 9283021.f1.doc]

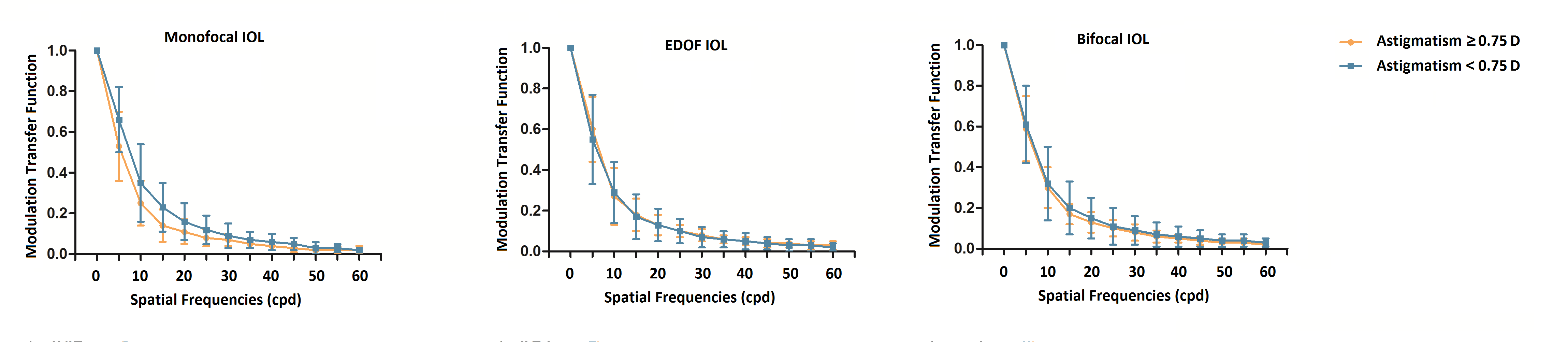
FIGURE S1: Comparison of the mean total (ocular) modulation transfer function (MTF) values between the two astigmatism subgroups of the 3 intraocular lens (IOL) groups. No significant difference was observed for any of the spatial frequencies. Bars around datapoints correspond to the standard deviation (SD) (total: indicates data calculated from total aberrations; EDOF = extended range of vision).


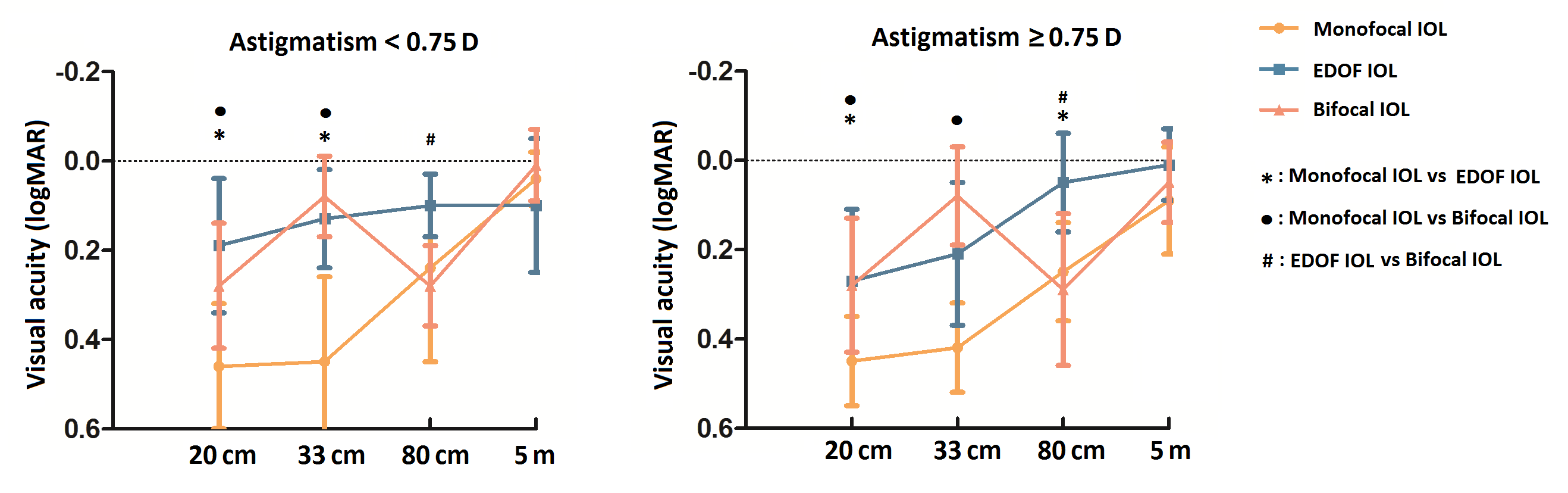


FIGURE S2: Comparison of the mean uncorrected visual acuities at all distances among the 3 intraocular lens (IOL) groups. Bars around datapoints correspond to the standard deviation (SD) (* = statistically significant difference between the monofocal group and the extended range of vision (EDOF) group [P<0.05]; ^●^ = statistically significant difference between the monofocal group and the bifocal group [P<0.05]; ^#^ = statistically significant difference between the EDOF group and the bifocal group [P<0.05]; logMAR= logarithm of minimum angle of resolution).


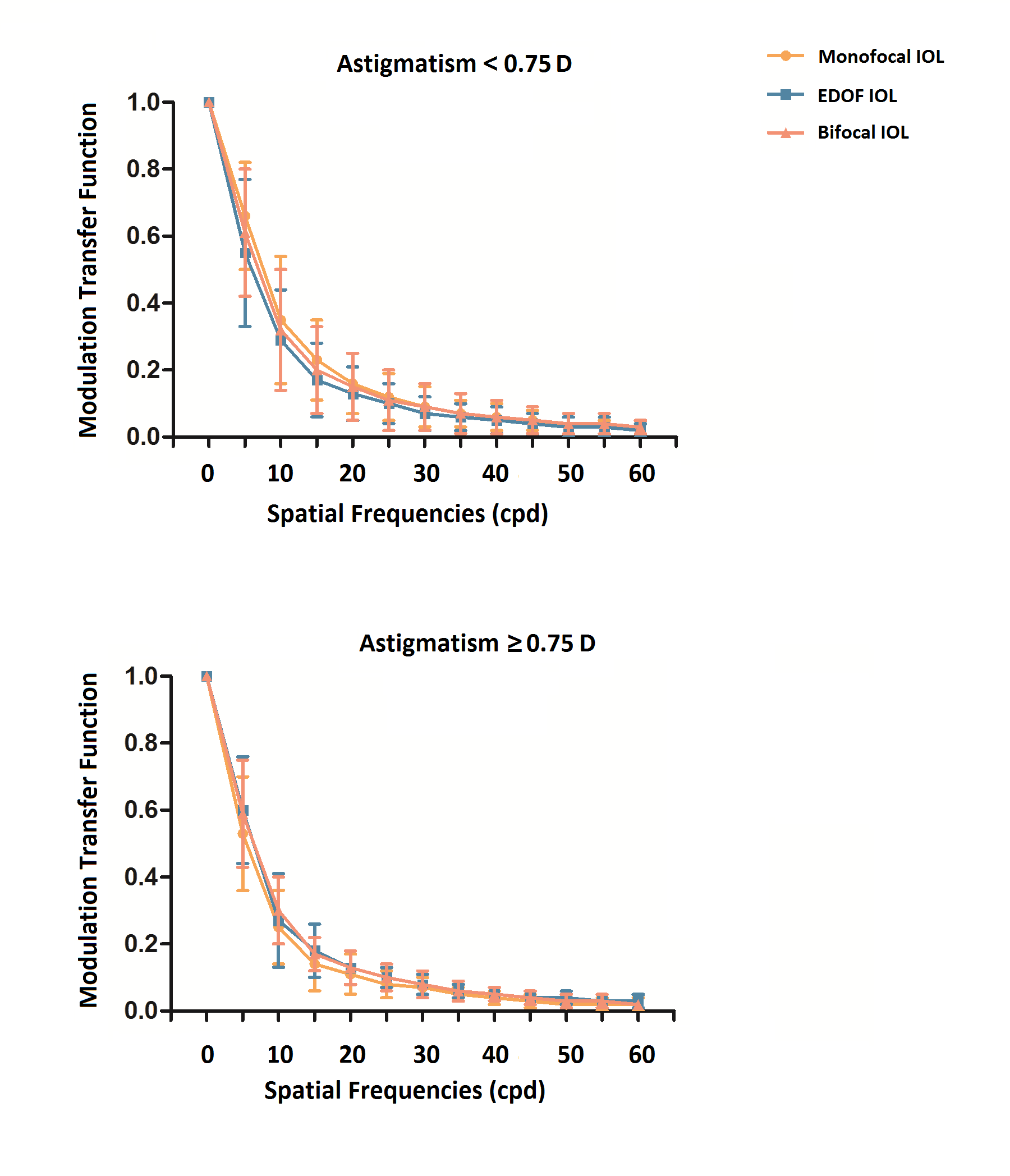


FIGURE S3: Comparison of the mean total (ocular) modulation transfer function (MTF) values among the 3 intraocular lens (IOL) groups. No significant difference was observed among the 3 IOL groups for any of the spatial frequencies. Error bars represent the standard deviation (SD) of the mean (total: indicates data calculated from total aberrations; EDOF = extended range of vision).

**TABLE S1: Intragroup comparison of objective optical quality outcomes**

| Parameter | Monofocal IOL | EDOF IOL | Bifocal IOL | P Value |
| --- | --- | --- | --- | --- |
| AR of MTF (total) (%) |  |  |  | 0.913 |
| Mean ± SD | 38.51±13.50 | 37.87±12.16 | 39.54±13.68 |  |
| Median (25% and 75% IR) | 34.20 (27.83, 47.50) | 39.40 (27.25, 47.68) | 37.00 (29.58, 46.88) |  |
| AR of MTF (HO) (%) |  |  |  | 0.074 |
| Mean ± SD | 73.47±21.80 | 64.14±15.06 | 60.78±15.85 |  |
| Median (25% and 75% IR) | 73.85 (57.88, 88.98) | 61.30 (53.45, 70.95) | 59.40 (51.33, 67.08) |  |
| SR of PSF (total) |  |  |  | 0.836 |
| Mean ± SD | 0.10±0.07 | 0.10±0.07 | 0.11±0.09 |  |
| Median (25% and 75% IR) | 0.08 (0.05, 0.16) | 0.09 (0.04, 0.15) | 0.10 (0.05, 0.12) |  |
| SR of PSF (HO) |  |  |  | 0.141 |
| Mean ± SD | 0.37±0.21 | 0.28±0.14 | 0.25±0.13 |  |
| Median (25% and 75% IR) | 0.36 (0.20, 0.51) | 0.26 (0.20, 0.32) | 0.23 (0.18, 0.29) |  |

Total: indicates data calculated from total aberration, HO: indicates data calculated from only high order aberration, IOL = intraocular lens; EDOF = extend depth-of-focus, AR = area ratio, MTF = modulation transfer function, SR = Strehl ratio, PSF = point spread function, SD = standard deviation, IR = interquartile ranges.

| Parameter | Monofocal IOL | | |  | EDOF IOL | | |  | Bifocal IOL | | |
| --- | --- | --- | --- | --- | --- | --- | --- | --- | --- | --- | --- |
|  | <0.75 D  (11 eyes) | ≥0.75 D  (9 eyes) | P value |  | <0.75 D  (10 eyes) | ≥0.75 D  (10 eyes) | P value |  | <0.75 D  (11 eyes) | ≥0.7 5 D  (9 eyes) | P value |
| AR of MTF (total ) (%) | 42.68±14.95 | 33.41±10.01 | 0.230 |  | 36.79±14.49 | 38.94±9.99 | 0.579 |  | 40.78±17.07 | 38.01±8.68 | 1.000 |
| SR of PSF (total) | 0.12±0.09 | 0.08±0.05 | 0.412 |  | 0.10±0.08 | 0.09±0.06 | 0.971 |  | 0.12±0.12 | 0.09±0.04 | 0.824 |
| Total VF-14 score | 77.99±12.27 | 79.51±6.37 | 0.766 |  | 95.73±5.34 | 95.02±6.03 | 0.912 |  | 93.02±7.80 | 90.78±6.43 | 0.552 |
| VF-14 (distance vision) | 95.26±8.24 | 95.11±7.97 | 0.766 |  | 99.64±1.13 | 98.57±3.45 | 0.684 |  | 96.27±6.21 | 97.57±3.43 | 1.000 |
| VF-14 (near vision) | 62.77±17.47 | 64.42±10.76 | 0.656 |  | 92.50±9.44 | 91.37±9.64 | 0.739 |  | 89.77±10.10 | 84.59±9.86 | 0.261 |
| Spectacle dependence | 2.09±1.22 | 2.00±0.87 | 1.000 |  | 3.60±0.70 | 3.30±0.82 | 0.436 |  | 3.64±0.50 | 3.44±1.01 | 1.000 |
| Glare | 3.36±0.92 | 3.33±0.71 | 0.766 |  | 3.30±0.82 | 3.40±0.84 | 0.796 |  | 2.91±1.14 | 2.78±1.30 | 0.824 |
| Halos | 3.36±0.81 | 4.00±0.00 | 0.095 |  | 3.60±0.70 | 3.60±0.52 | 0.853 |  | 2.91±1.22 | 3.67±0.71 | 0.175 |
| Satisfaction score | 3.36±0.92 | 3.56±0.53 | 0.656 |  | 4.20±0.63 | 4.30±0.67 | 0.739 |  | 4.45±0.52 | 3.22±0.83 | 0.002* |

TABLE S2: Comparison of objective optical quality and visual function questionnaire scores between the 2 astigmatism subgroups in the 3 IOL groups

Total: indicates data calculated from total aberration, IOL = intraocular lens, EDOF = extend depth-of-focus, AR = area ratio, MTF = modulation transfer function, SR = Strehl ratio, PSF = point spread function, VF-14 = Visual Function Index-14.

The results are expressed as mean ± standard deviation.

*Statistically significant (P<0 .05).

TABLE S3: Comparison of objective optical quality and visual function questionnaire scores among the 3 IOL types with various preoperative cornea astigmatism

| Parameter | Astigmatism <0.75 D | | | |  | Astigmatism ≥0.75 D | | | |
| --- | --- | --- | --- | --- | --- | --- | --- | --- | --- |
|  | Monofocal IOL  (A) | EDOF IOL  (B) | Bifocal IOL  (C) | P value |  | Monofocal IOL  (A) | EDOF IOL  (B) | Bifocal IOL  (C) | P value |
| AR of MTF (total) (%) | 42.68±14.95 | 36.79±14.49 | 40.78±17.07 | 0.660 |  | 33.41±10.01 | 38.94±9.99 | 38.01±8.68 | 0.374 |
| SR of PSF (total) | 0.12±0.09 | 0.10±0.08 | 0.12±0.12 | 0.781 |  | 0.08±0.05 | 0.09±0.06 | 0.09±0.04 | 0.826 |
| Total VF-14 score | 77.99±12.27 | 95.73±5.34 | 93.02±7.80 | 0.001*  A-B 0.002*  A-C 0.016*  B-C 1.000 |  | 79.51±6.37 | 95.02±6.03 | 90.78±6.43 | 0.001*  A-B <0.001*  A-C 0.018*  B-C 1.000 |
| VF-14 (distance vision) | 95.26±8.24 | 99.64±1.13 | 96.27±6.21 | 0.248 |  | 95.11±7.97 | 98.57±3.45 | 97.57±3.43 | 0.285 |
| VF-14 (near vision) | 62.77±17.47 | 92.50±9.44 | 89.77±10.10 | <0.001*  A-B 0.001*  A-C 0.005*  B-C 1.000 |  | 64.42±10.76 | 91.37±9.64 | 84.59±9.86 | <0.001*  A-B <0.001*  A-C 0.020*  B-C 0.806 |
| Spectacle dependence | 2.09±1.22 | 3.60±0.70 | 3.64±0.50 | 0.003*  A-B 0.011*  A-C 0.011*  B-C 1.000 |  | 2.00±0.87 | 3.30±0.82 | 3.44±1.01 | 0.006*  A-B 0.030*  A-C 0.009*  B-C 1.000 |
| Glare | 3.36±0.92 | 3.30±0.82 | 2.91±1.14 | 0.570 |  | 3.33±0.71 | 3.40±0.84 | 2.78±1.30 | 0.525 |
| Halos | 3.36±0.81 | 3.60±0.70 | 2.91±1.22 | 0.355 |  | 4.00±0.00 | 3.60±0.52 | 3.67±0.71 | 0.132 |
| Satisfaction score | 3.36±0.92 | 4.20±0.63 | 4.45±0.52 | 0.009*  A-B 0.108  A-C 0.009*  B-C 1.000 |  | 3.56±0.53 | 4.30±0.67 | 3.22±0.83 | 0.010*  A-B 0.144  A-C 1.000  B-C 0.009* |

Total: indicates data calculated from total aberration, IOL = intraocular lens; EDOF = extend depth-of-focus, AR = area ratio, MTF = modulation transfer function, SR = Strehl ratio, PSF = point spread function, VF-14 = Visual Function Index-14.

The results are expressed as mean ± standard deviation.

*Statistically significant (P <0 .05).
